# Electromagnetic Transmitter-Based Prostate Gating for Dose-Escalated Linac-Based Stereotactic Body Radiation Therapy: An Evaluation of Intrafraction Motion

**DOI:** 10.3390/curroncol31020072

**Published:** 2024-02-09

**Authors:** Berardino De Bari, Geoffroy Guibert, Sabrine Slimani, Yanes Bashar, Terence Risse, Nicole Guisolan, Juliane Trouillot, Jonathan Abel, Patrick Weber

**Affiliations:** 1 Radiation Oncology Department, Réseau Hospitalier Neuchâtelois, CH-2300 La Chaux-de-Fond, Switzerlandnicole.guisolan@rhne.ch (N.G.);; 2 Medical Physics Department, Réseau Hospitalier Neuchâtelois, CH-2300 La Chaux-de-Fond, Switzerland

**Keywords:** prostate cancer, stereotactic body radiation therapy (SBRT), extreme hypofractionation, image-guided radiation therapy (IGRT), intrafraction motion mitigation, real-time electromagnetic tracking

## Abstract

Background: Stereotactic Body Radiotherapy (SBRT) is as a standard treatment for prostate cancer (PCa). Tight margins and high dose gradients are needed, and the precise localization of the target is mandatory. Our retrospective study reports our experience regarding the evaluation of intrafraction prostate motion during LINAC-based SBRT evaluated with a novel electromagnetic (EM) tracking device. This device consists of an integrated Foley catheter with a transmitter connected to a receiver placed on the treatment table. Methods: We analyzed 31 patients who received LINAC-based SBRT using flattening filter-free (FFF) volumetric modulated arc therapy (VMAT). The patients were scheduled to be treated for primary (*n* = 27) or an intraprostatic recurrent PCa (*n* = 4). A simulation CT scan was conducted while the patients had a filled bladder (100–150 cc) and an empty rectum, and an EM tracking device was used. The same rectal and bladder conditions were employed during the treatment. The patients received 36.25 Gy delivered over five consecutive fractions on the whole prostate and 40 Gy on the nodule(s) visible via MRI, both delivered with a Simultaneous Integrated Boost approach. The CTV-to-PTV margin was 2 mm for both the identified treatment volumes. Patient positioning was verified with XVI ConeBeam-CT (CBCT) matching before each fraction. When the signals exceeded a 2 mm threshold in any of the three spatial directions, the treatment was manually interrupted. A new XVI CBCT was performed if this offset lasted >20 s. Results: We analyzed data about 155 fractions. The median and mean treatment times, calculated per fraction, were 10 m31 s and 12 m44 s (range: 6 m36 s–65 m28 s), and 95% of the fractions were delivered with a maximum time of 27 m48 s. During treatment delivery, the mean and median number of XVI CBCT operations realized during the treatment were 2 and 1 (range: 0–11). During the treatment, the prostate was outside the CTV-to-PTV margin (2 mm), thus necessitating the stoppage of the delivery +/− a reacquisition of the XVI CBCT for 11.2%, 8.9%, and 3.9% of the delivery time in the vertical, longitudinal, and lateral direction, respectively. Conclusions: We easily integrated an EM-transmitter-based gating for prostate LINAC-based SBRT into our normal daily workflow. Using this system, a 2 mm CTV-to-PTV margin could be safely applied. A small number of fractions showed a motion exceeding the predefined 2 mm threshold, which would have otherwise gone undetected without intrafraction motion management.

## 1. Introduction

Modern radiobiological models and available clinical studies strongly support the assumption that prostate cancer (PCa) has a linear quadratic α/β ratio lower than that in the majority of other human tumors (estimated to be ~1.5 Gy vs. 5–10 in other tumor types). Accordingly, PCa could benefit from hypofractionated regimens of radiation therapy (RT) [1,2,3,4,5]. Based on the recent major technological advances, RT can now be delivered with better organs-at-risk (OARs) sparing and prostate irradiation. Moreover, these improvements have also allowed the introduction of extreme hypofractionation using stereotactic body radiation therapy (SBRT) for PCa: even after long follow-up times, this therapeutic approach showed optimal results both in terms of biochemical control and side effects [6,7]. Two large systematic reviews [8,9], one of which was of a phase III study [10], clearly support the adoption of SBRT for PCa. Unfortunately, the efficacy data of the PACE-B trial are still pending [11].

High dose gradients and the narrowest margins are employed in SBRT. In this context, precision in dose delivery is even more crucial than in conventionally fractionated RT in order to avoid administering an overdose to the OARs and/or suboptimal target coverage. Intrafraction prostate motion, mainly occurring due to rectal and bladder filling, is a major issue with respect to PCa SBRT [12,13,14]. Lovelock et al. already showed that without the implementation of a strategy of continuous monitoring and intervention, prostate intrafractional motion can lead to target missing for approximately 10% of patients [15]. Based on this background, the importance of implementing robust strategies for image-guided radiation therapy (IGRT) to obtain precise targeting and gating and/or tracking of a target is clear. Usually, such strategies are based on the implantation of intraprostatic fiducials. One of the limits of these devices is that they are radiopaque and interfere, via their artifacts, with the quality of imaging modalities (e.g., prostatic MRI) that could be used for the staging and restaging of patients.

In this retrospective study, we report our experience with a novel electromagnetic (EM)-GPS-transmitter-based device consisting of an integrated Foley catheter with a transmitter connected to a receiver placed on a treatment table. We decided to adopt this system as it could be adopted without any surgical intervention or definitive implantation of intraprostatic coils [16]. In particular, we report the results in terms of the real- time tracking of prostate organ motion during dose-escalated LINAC-based PCa SBRT. Early clinical data on the tolerance for the device and the early toxicity data of the patients are also reported.

## 2. Material and Methods

### 2.1. Patient Setup and Treatment Planning

All the patients underwent a simulation CT scan in a supine position with their arms over their chests. A ProSTEP ABS immobilization system (Innovative Technologie Völp, Innsbvruck, Austria) fixed to a couch was adopted in order to ensure ankle fixation. By using a 16 French Foley catheter, we filled the bladder with 100–150 cc of saline solution, thus ensuring good reproducibility of bladder filling. A rectal micro-enema was administered 15–30 min before the simulation CT scan in order to void the rectum. We did not use rectal spacer devices, and no rectal immobilization was induced. The same rectal and bladder statuses were obtained before each radiotherapy fraction.

The delineation of the prostate was performed after a co-registration of the simulation CT scan and T2- weighted prostate MRI.

Based on the study by Herrera et al. [17], the treatment volumes were defined as follows:

- PTV1: Whole prostate +/− distal 1.5 cm of seminal vesicles (only in ISUP 3–5 patients [18]) +2 mm;
- PTV2: GTV(s) defined through MRI of +2 mm.

The PTV1 group received 36.25 Gy over 5 consecutive fractions of 7.25 Gy/fraction, while the PTV(s) group received 40 Gy over 5 consecutive fractions of 8 Gy/fraction, delivered with a Simultaneous Integrated Boost approach.

The rectum (contoured from the first slide below the sigmoid to the anal margin), the bladder, the femoral heads (contoured until the first slide below the whole lesser trochanter), and the urethra were the organs at risk that were defined. We added a margin of 2 mm around the urethra to obtain a planning-organ-at-risk volume (PRV) for the urethra, and a urethra-sparing approach was adopted. The presence of the catheter clearly helped the radiation oncologist in the definition of the urethra, as it was easily visible in the CT images.

The following dose constraints were applied to the OARs [17,18]:

- Rectum—V27 Gy < 20 cc, V13.5 < 30%, V6.7 Gy < 60% and max dose 40.5 Gy;
- Bladder—V19 Gy < 15 cc, V10.6 Gy < 30%;
- Urethra—V39 Gy < 1 cc and V41 Gy < 0.1 cc;
- Femoral heads—30 Gy < 10 cc.

We treated our patients with 6 MV flattening-filter-free (FFF) single-arc volumetric modulated arc therapy (VMAT) applied using a VersaHD linear accelerator (Elekta AB, Stockholm, Sweden). Plans were optimized to have at least 100% of the PTV covered by the 95% isodose and to fulfill the dose–volume constraints on the OARs (see above). The treatment plans were calculated using a CC convolution algorithm included in the Pinnacle 16.2.1 Treatment Planning System (Philips, Eindhoven, The Nederlands). Pinnacle 16.2.1 allows the entry of the desired delivery time as a parameter and, after optimization, shows an estimated delivery time. There were no differences in the treatment volumes and doses amongst primary and relapsing patients.

### 2.2. Intrafraction Motion Tracking and Intervention Protocol

Intrafraction organ motion was estimated by using the Raypilot System (Micropos Medical AB, Gothenburg, Sweden) [19,20]. This is a new real-time EM tracking device based on a wired transmitter integrated into a dedicated lumen of the Raypilot Hypocath, which is a Foley catheter that is implanted into a patient before the beginning of a treatment. Signals from the transmitter are received by the Raypilot Receiver, a platform that is placed under the patient on an existing carbon fiber treatment couch. The Raypilot System was left in the urethra for 5 days from the time of the first treatment to the end of the last treatment.

The transmitter consists of a choke coil (with a length of 10 mm and a diameter of 3 mm) and a cable that is connected to the RayPilot Receiver before and during each fraction to activate the device. The signal generated by the transmitter is captured by an antenna array, allowing for the identification of the position of the transmitter. Raypilot System is calibrated to the isocenter of the treatment room, thus allowing treatment localization and motion tracking. The software returns information about the prostate’s position along three-dimensional axes (lateral, longitudinal, and vertical) at a sampling frequency of 30 Hz. Notably, yaw (vertical axis rotations) and pitch (lateral axis rotations) are also detected by this system. Before treatment delivery, we measured and took into account treatment couch bending due to patient weight. After the positioning of the catheter, the EM transmitter was positioned roughly in the middle of the prostate.

**Figure 1:**
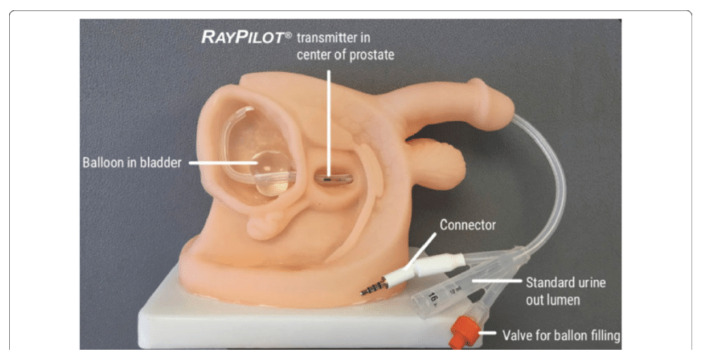
A picture showing the final installation in a patient.

Figure 1 provides an idea of the installation of the system in a patient.

The setup of the patients was verified using Elekta X-ray volume imager (XVI) ConeBeam-CT (CBCT) soft tissue-based matching before treatment delivery. Notably, in order to reduce the time taken for XVI CBCT acquisition, we optimized the IGRT setup (M15 XVI version 5.0.2 b72) and modified the gantry speed parameter up to 360°/min. All the other parameters (kV, frames, mA/frame, etc.) were not changed. This allowed a reduction in the acquisition time by a factor two (approximately 1 min instead of 2 min). Before clinical implementation, this IGRT setup was tested and approved on phantoms (i.e., an MIMI phantom cube or an anthropomorphic phantom from the AVA ATOM^®^ phantom family CIRS for anatomical pelvic localization). The phantoms were scanned, centered, and marked using a radiopaque fiducial marker; installed on a precise table; and aligned with the LINAC lasers.

The start of motion tracking was the beginning of the acquisition of the XVI CBCT. The initial position detected with the system was set equal to zero. Prostate motion was evaluated by using all shifts in the transmitter position as surrogates for prostate motion.

We manually interrupted the beam delivery when the shift of the transmitter exceeded the CTV to PTV margins (2 mm) in any of the three spatial directions. In our protocol, a new XVI CBCT was acquired and matched when the drift outside this 2 mm tolerance lasted >20 s; additionally, a new Raypilot position was set, and the treatment beam was resumed.

### 2.3. Data Acquisition, Processing, and Analysis

The Raypilot software tools (version 4.2.91) allowed the automatic measurement and recording of the real-time transmitter displacements for each treatment fraction by using. The evaluation of the transmitter trajectories of the gated treatments has been described previously [19]. After the treatment, the log files including the transmitter positions and beam-on indications were exported with an update rate of 25 Hz in XML format. The Raypilot software generates an excel file containing information about the position of the transmitter every 25 Hz. Data were analyzed with Matlab (Vers. R2022b) in order to extract information such as duration, number of XVI CBCT procedures, and transmitter displacement during the different phases of the treatment (setup, delivery, and setup + delivery).

### 2.4. Data about Treatment Duration

Several data analysis approaches were used:

- Per-patient, realized including all the 5 delivered sessions of the total treatment;
- Per-fraction, including all the patients, in order to determine whether there is a possible influence of the session number on treatment duration;
- Per-session, including data on all the sessions and on all the patients.

**Figure 2:**
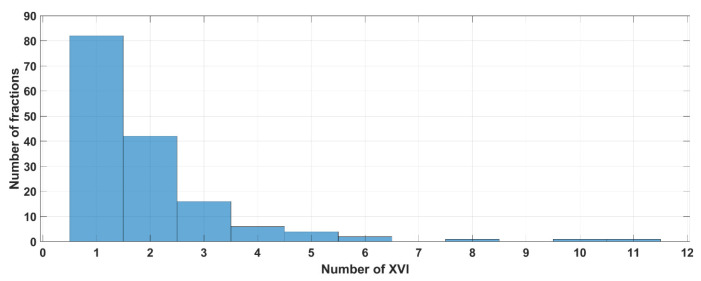
Distribution of the number of XVI procedures performed during the fraction (total *n* = 155).

In our analysis, we also identified 2 moments: the setup phase and the treatment phase (Figure 2). The setup phase included the alignment of a patient, corrections of table position, gantry rotation for XVI CBCT acquisition, achievement of XVI, and registration of the table. Treatment phase started when the table had been moved into the correct treatment position and the patient was ready for treatment, and it included the rotation of the gantry for beam delivery and the delivery of beam irradiation. XVI CBCT performed during irradiation is classified/added in the setup phase.

## 3. Results

From 24.01.2022 to 02.10.2022, we treated 31 patients using the Raypilot Hypocath in order to deliver a prostate SBRT for a primary (*n* = 27) or an intraprostatic recurrence of PCa (*n* = 4). All patients were N0 and M0 at the standard staging procedures. Following local Institutional protocols, all the patients were subjected to pre-treatment MRI. Patients with relapsing tumors also received a restaging with a [^68^Ga]- or [^18^F]-labeled prostate-specific membrane antigen (PSMA) PET/CT, and intraprostatic relapse was documented with targeted biopsies. Table 1 summarizes the features of the patients.

Finally, 155 treatment fractions were delivered, and 298 XVI CBCT-to-planning CT matchings were performed. These are the data analyzed and reported in this study.

### 3.1. Data about XVI CBCT Acquisition

Figure 2 and Table S1 (see Supplementary Material) show the results of the analysis of the number of the XVI CBCT images acquired before and/or during SBRT delivery. Looking at the whole population, the median number of XVI CBCT images acquired over the entire treatment time, during the setup phase, and during the delivery phase wad 1, 1, and 0 (see Table S1). Looking at all the fractions (155), 77% of them (120/155) required one or two XVI CBCT procedures (Figure 2).

Only one session needed 11 XVI CBCT procedures, of which 6 were performed during setup and 5 were conducted during irradiation phases (Table S1 last column). One patient session required 10 XVI CBCT procedures before irradiation without any interruption during the delivery.

**Figure 3:**
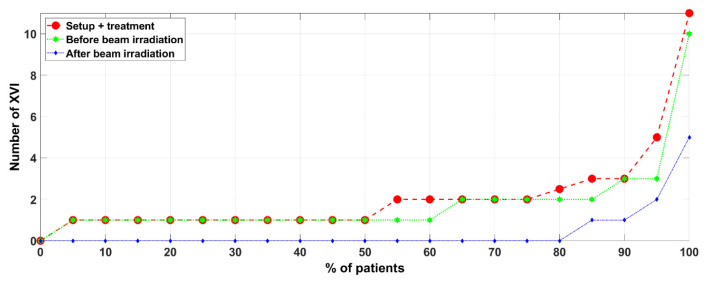
Percentage of patients (X-axis) as a function of the number of XVI (Y-axis) procedures for 31 patients or sessions of treatment (total *n* = 155).

Globally, up to 90% of the fractions needed three XVI CBCT procedures or less throughout the whole treatment period (setup + delivery time, see Figure 3).

Table 2 shows data about the number of XVI CBCT images evaluated per treatment session.

### 3.2. Results of the Per-Patient Analysis

This per-patient analysis was initially realized on the whole population. Then, we also identified three subgroups of patients:

- *Group A* (3 patients, 15 sessions)—only one setup control was performed and needed before the beginning of the treatment, without any other setup verification required during either the setup phase or the delivery phase.
- *Group B* (15 patients, 75 sessions)—at least one setup control was performed and needed before the beginning of the treatment. No other setup controls were needed during the delivery phase. This group encompasses Group A.
- *Group C* (16 patients, 80 sessions)—at least one supplementary setup control was performed and needed during the delivery of the treatment. No other setup controls were needed during the setup phase.

**Figure 4:**
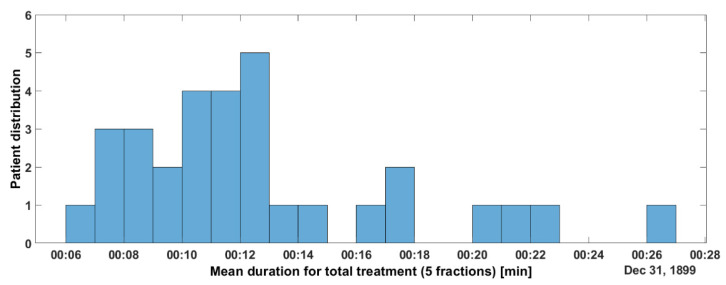
Distribution of the patients according to the mean duration of their total treatment time (established on the 5 treatment sessions).

Figure 4 and Table S2 (see Supplementary Material) summarize the data regarding the per-patient analysis: the patient distribution was calculated according to the patients’ mean total treatment time (established over the five sessions). It is interesting to note that the mean duration of the fraction was ≥18 min for 90% of the patients.

**Figure 5:**
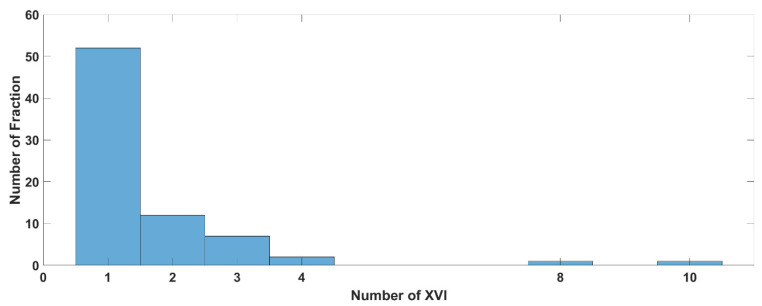
Distribution of the number of XVI CBCT procedures in Group B (*n* = 15).

**Figure 6:**
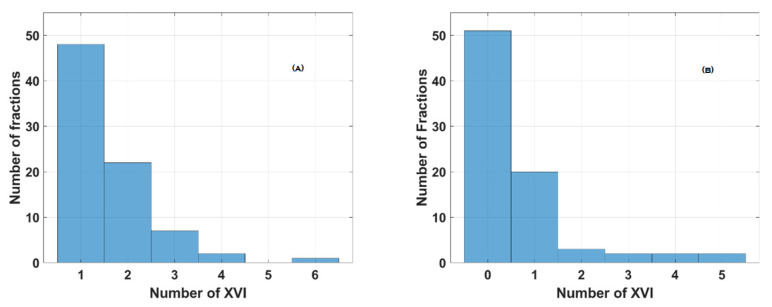
Distribution of the number of XVI CBCT procedures in Group C (*n* = 16) before (**A**) and during irradiation (**B**).

Figure 5 and Figure 6A,B and Table S3 (see Supplementary Material) summarize the data about the number of XVI CBCT procedures performed in groups B and C, respectively. In Group B, 60/75 (80%) fractions needed only one or 2 XVI CBCTs (Figure 5).

### 3.3. Results of the Per-Session Analysis

**Figure 7:**
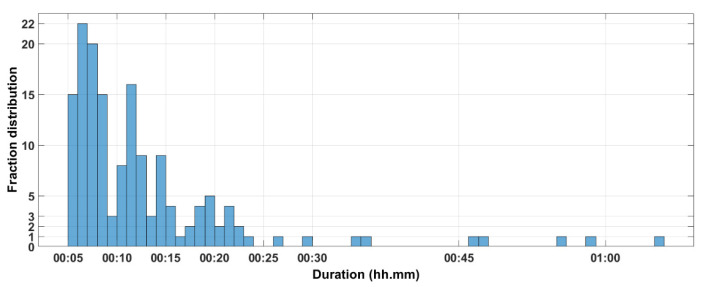
Distribution of the fraction duration for the 155 sessions.

Figure 7 summarizes the data regarding the per-session analysis. We found that 94% of the fractions lasted less than 25 min and that more than 50% of the fractions were delivered in less than 11 min.

### 3.4. Prostate Displacements (% of Time Outside the CTV-to-PTV Margin of 2 mm)

**Figure 8:**
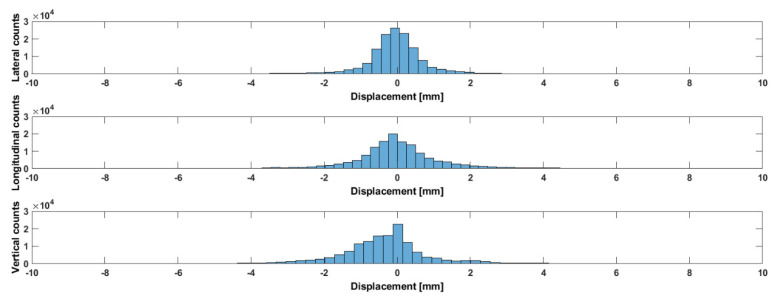
Distribution of real prostate motion (laterally, longitudinally, and vertically) for 31 patients with respect to 155 fractions during setup and treatment.

Table 3 and Figure 8 show data about prostate motion in the three spatial directions. This figure clearly shows that the three-dimensional prostate translational shifts are asymmetrical.

In our analysis, we were interested in verifying if the CTV-to-PTV margins (2 mm) that we applied according to the available literature [16] were safe enough in our daily clinical practice. For this reason, we calculated the amount of time we were obliged to stop the treatment during its delivery because of a displacement of the target outside the given margin (before deciding whether to restart the treatment or redo the CBCT procedure).

Table 4 shows the results in terms of the % of time the CTV was outside the CTV-to-PTV margin (2 mm) for the whole population and for the three identified groups of patients.

### 3.5. Results of the Per-Fraction Analysis

Table 5 summarizes the data regarding the per-fraction analysis.

### 3.6. Clinical Compliance

All patients reported, during the treatment, G1-2 dysuria and/or pollakyuria related to the presence of the catheter. All the patients reported the regression of these symptoms when the catheter was removed at the moment of the last fraction of radiotherapy.

Following our local protocol, all the patients (30–40 patients) had follow-up visits after the end of radiotherapy: during these visits, 3/31 patients (9.6%) reported contracting a urinary infection after the end of radiotherapy, which was treated with 7–10 days of antibiotics and resolved at the moment of the visit for all of the patients.

## 4. Discussion

To the best of our knowledge, this is the largest study reporting data on intrafraction prostate motion during LINAC-based SBRT evaluated with a novel electromagnetic (EM) tracking device consisting of an integrated Foley catheter with a transmitter connected to a receiver placed on a treatment table. We confirm, based on a larger population, the technical and clinical feasibility of such an approach, as already shown by Panizza et al. [16].

Nevertheless, we consider that some further interesting, hypothesis-generating data should be discussed.

The available literature already clearly supports the daily adoption of IGRT protocols, as they improve treatment accuracy and reduce side effects associated with prostate irradiation [21,22,23,24]. Regarding PCa irradiation, IGRT can be realized by using several technical solutions and strategies: XVI CBCT, orthogonal radiographs with or without intraprostatic fiducials, XVI CBCT, and ultrasound-based approaches. All these solutions are efficient enough to take into account interfraction motion [24]. Nevertheless, these approaches allow the verification of the initial patient setup, but most of them could not assess and correct intrafraction organ motion. Prostate SBRT requires high precision, both in terms of MRI-based contouring and in terms of dosimetry (very-sharp dose gradients, the adoption of FFF-VMAT beams, and urethra-sparing approaches). Thus, it is easy to understand the importance of an accurate tracking of the position of a target during the throughout the fraction; for these reasons, any real-time tracking/gating solution should be preferred in order to detect and correct any potential intrafraction target displacement.

The implementation of the Raypilot System allowed not only the online tracking of the prostate but also the monitoring of its behavior in terms of intrafractional motion; in this sense, our data favorably compare with those previously published [15,25,26,27,28,29].

In particular, we confirm that the predominant motion was in the vertical (antero-posterior) direction, and we also show that the prostate could exhibit some intrafraction lateral displacements.

Our study also confirms the importance of the continuous monitoring of prostate position and intervention (CMI) when a tight 2 mm margin from the CTV to the PTV is adopted during PCa SBRT [15,29,30,31,32,33,34]. According to our findings, the vast majority of the patients would have been correctly treated without any intrafractional adjustment with a CTV-to-PTV margin of 5 mm, but wider margins would not allow dose escalation for the prostate and/or a reduction in the dose administered to the OARs. The integration of online shift monitoring allows the adoption of tighter-than-conventional margins with adequate target coverage.

In our study, the Raypilot Hypocath allowed the identification and correction of shifts that would have otherwise remained unnoticed and unaccounted for. These potentially undetected displacements of more than 2 mm finally accounted for up to 26% of the treated fractions (Table 4). Notably, these displacements occurred despite the very strict bowel and bladder preparations that we adopted during the acquisition of the simulation CT-scan and before each treatment’s setup and delivery.

Several commercially available solutions allow continuous intrafraction tracking of the prostate during treatment [24,35]. In the evaluation of these devices, their efficacy should be balanced against their cost-effectiveness: for example, several of them are expensive, requiring additional equipment unavailable on a standard LINAC or, in the most expensive cases, the implementation of new, complex machines, like the CyberKnife (CK) robotic radiosurgery system or the MRI-LINAC. Notably, most of these techniques also entail long treatment times ranging from 20 to 90 min and/or only allow the treatment of a small number of patients/day. These are important limitations on the implementation of these techniques, particularly in tertiary care institutions like ours.

The Raypilot Solution is a non-ionizing real-time positioning system. This solution exhibits three important features: 1. It can be completely removed after treatment completion; this means that it would not interfere in an MRI-based follow-up, 2. It does not require any permanent treatment room installations, thus providing cost-effectiveness advantages over the available options 3. It does not interfere either with the acquisition of XVI CBCT images or the delivery of the treatment [25,26]. Moreover, when compared to transperineal implanted wired transmitters, the introduction of the Raypilot HypoCath resulted in a less-invasive and more stable solution [27,28].

Nevertheless, we recommend combining real-time prostate motion monitoring via Raypilot with an independent IGRT system, namely, XVI CBCT, to account for optimal rectal and bladder filling. The reason for this is that the absolute localization accuracy of the system may not be high enough for the interfraction localization of the prostate, mostly due to the uncertain positional reproducibility of the catheter balloon with respect to the bladder wall.

Looking at the potential impact of the Raypilot System on the organizational aspects of a radiation oncology department, our data show that, even when the treatment was interrupted for target motion correction, 94% of the fractions were delivered in less than 25 min, and more than 50% of the fractions were delivered in less than 11 min. We can conclude that such a solution could be quite easily integrated into a daily radiotherapy workflow.

This finding is noteworthy, as longer treatments may increase patient discomfort, thus potentially affecting intrafraction motion and increasing the risk of errors; all these aspects negatively affect the clinical benefits associated with SBRT [32,33]. Moreover, we also see that in our experience, the setup phase contributes to 75% of the total treatment time. These data reflect the need for a higher number of XVI procedures in the setup phase when compared to the treatment phase and validate the interest in implementing all the strategies to reduce the acquisition time for the XVI. Our innovative protocol of XVI acquisition goes in this direction.

Lat but not least, we should acknowledge, in our study, that we present data on quite a small population and that we do not present clinical data (only data about target displacements are presented).

## 5. Conclusions

We successfully implemented an EM-based real-time tracking strategy during dose-escalated PCa SBRT. Most of the patients presented shifts that were <2 mm in any direction. Continuous motion monitoring allowed the identification and correction of the small number of fractions presenting larger shifts.

## Figures and Tables

**Table 1:** Clinical features of the patients enrolled in this study (data on 31 patients for 155 sessions).

	Number of Cases	%
Number of patients	31	100
Age at the moment of the diagnosis (years)		
Mean	73.6	
Median	73.8	
Range (min–max)	(60.7–84.6)	
T stage (defined with pre-treatment MRI)		
1c	1	3.2
2a	15	48.3
2b	3	9.7
2c	5	16.1
3a	3	9.7
Relapsing pre-treated PCa	4	13
PSA at the moment of the diagnosis (ng/mL) *		
Mean	8.57	
Median	8.43	
Range (min–max)	(3.05–20)	
ISUP category risk (as defined in [21])		
1	5	16.1
2	13	42
3	7	22.6
4	2	6.4
5	0	0
Relapsing pre-treated PCa	4	12.9

Legend: MRI = Magnetic Resonance Imaging; PSA = Prostate-Specific Antigen; ISUP = International Society of Urological Pathology. * For the 4 patients with an intraprostatic relapse, we report data acquired at the moment of the relapse.

**Table 2:** Total, median, minimum, and maximum number of XVI CBCT images evaluated per treatment session (31 patients).

	Total *n*. of XVI CBCT	Median	Min	Max
**Session 1**	62	1	1	11
**Session 2**	60	2	1	5
**Session 3**	57	1	1	6
**Session 4**	70	1	1	10
**Session 5**	50	1	1	5

**Table 3:** Mean, standard deviation (SD), and absolute mean of the real prostate data from 155 fractions (setup and treatment). The negative sign indicates a displacement in right, inferior, and posterior directions.

Direction	Mean (mm)	SD (mm)	Mean Absolute (mm)
**Lateral**	−0.38	2.76	1.0969
**Longitudinal**	−0.28	2.42	1.208
**Vertical**	−0.38	1.92	1.0957

**Table 4:** Time spent in % of time outside the CTV-to-PTV margin for the whole population and for Groups A, B, and C.

Group	Setup Phase			Delivery Phase			Overall Time (Setup + Delivery)		
	*lat*	*Lng*	*Vrt*	*Lat*	*Lng*	*Vrt*	*Lat*	*Lng*	*Vrt*
**Whole population**	12%	20%	22%	4%	9%	11%	10%	17%	19%
**Group A**	4%	2%	4%	0	0.2%	18%	2%	1%	7%
**Group B**	17%	25%	26%	2%	5%	9%	13%	19%	21%
**Group C**	9%	17%	19%	5%	12%	13%	8%	15%	17%

**Table 5:** Mean, median, minimum, and maximum durations for each fraction (*n* = 31 patients).

	Mean	Median	Min	Max
**Fraction #1**	14 min 33 s	8 min 48 s	5 min 49 s	1 h 05 min 28 s
**Fraction #2**	12 min 54	11 min 49	5 min 23 s	34 min 35 s
**Fraction #3**	12 min 36	10 min 53	5 min 31 s	47 min 15 s
**Fraction #4**	12 min 00	8 min 40	5 min 18 s	55 min
**Fraction #5**	11 min 32	8 min 39	5 min 21 s	58 min 28 s

## Data Availability

The data presented in this study are available on request from the corresponding author. The data are not publicly available due to Swiss patient privacy rules.

## Supplementary Materials

The following supporting information can be downloaded at https://www.mdpi.com/article/10.3390/curroncol31020072/s1. **Table S1.** Median, min and max number of XVI CBCT during the total treatment time, during the setup phase and during the irradiation phase (data on 31 patients, 155 sessions); **Table S2.** Mean value with SD, median, min and Max for total treatment duration in our “per patient” analysis. Legend: min = minutes; s = seconds.; **Table S3.** Median, Minimum, and Maximum number of XVI CBCT for the 3 identified Groups.
