# Supplementary material for: Electromagnetic Transmitter-Based Prostate Gating for Dose-Escalated Linac-Based Stereotactic Body Radiation Therapy: An Evaluation of Intrafraction Motion
Source: Curr Oncol. 2024 Feb 9;31(2):962–74. doi: 10.3390/curroncol31020072 (PMC10887766; doi:10.3390/curroncol31020072)
Supplement: Supplementary file 1 [file curroncol-31-00072-s001.zip › curroncol-2785033-supplementary.pdf]

Supplementary material

Table S1. Median, min and max number of XVI CBCT during the total treatment time, during the setup phase and during the irradiation phase (data on 31 patients, 155 sessions).

	Median	Min	Max
**Overall treatment (Setup + delivery)**	1	1	11
**Before delivery**	1	1	10
**During delivery**	0	0	5

Table S2. Mean value with SD, median, min and Max for total treatment duration in our "per patient" analysis. Legend: min = minutes ; s = seconds.

	Mean value	SD	Median	Min	Max
**Whole population**	12min44s	±9min39s	10min31s	5min18s	1h05min28s
**Group A**	8min10s	± 3min03s	7min48s	5min47s	18min11s
**Group B**	11min08s	± 9min19s	7min50s	5min18s	58min28s
**Group C**	14min12s	±9min47s	11min48s	5min41s	1h05min28s

Table S3. Median, Minimum, and Maximum number of XVI CBCT for the 3 identified Groups.

Group	Median	Minimum	Maximum
**Whole population**	1	1	11
**Group B**	1	1	10
**Group C, global treatment**	2	1	11
**Group C, setup phase**	1	1	6
**Group C, delivery phase**	0	0	5
